# Lack of efficacy of hydroxychloroquine and azithromycin in patients hospitalized for COVID-19 pneumonia: A retrospective study

**DOI:** 10.1371/journal.pone.0252388

**Published:** 2021-06-09

**Authors:** Anis Saib, Walid Amara, Pascal Wang, Simon Cattan, Azeddine Dellal, Kais Regaieg, Stephane Nahon, Olivier Nallet, Lee S. Nguyen

**Affiliations:** 1 Cardiology Department, Groupe Hospitalier Intercommunal Le Raincy-Montfermeil, Montfermeil, France; 2 Pneumology Department, Groupe Hospitalier Intercommunal Le Raincy-Montfermeil, Montfermeil, France; 3 Rheumatology Department, Groupe Hospitalier Intercommunal Le Raincy-Montfermeil, Montfermeil, France; 4 Intensive Care Medicine Department, Groupe Hospitalier Intercommunal Le Raincy-Montfermeil, Montfermeil, France; 5 Gastroenterology Department, Groupe Hospitalier Intercommunal Le Raincy-Montfermeil, Montfermeil, France; 6 Research & Innovation Department (RICAP), CMC Ambroise Paré, Neuilly-sur-Seine, France; University of Palermo, ITALY

## Abstract

**Background:**

Hydroxychloroquine combined with azithromycin (HCQ/AZI) has initially been used against coronavirus disease-2019 (COVID-19). In this retrospective study, we assessed the clinical effects of HCQ/AZI, with a 28-days follow-up.

**Methods:**

In a registry-study which included patients hospitalized for COVID-19 between March 15 and April 2, 2020, we compared patients who received HCQ/AZI to those who did not, regarding a composite outcome of mortality and mechanical ventilation with a 28-days follow-up. QT was monitored for patients treated with HCQ/AZI. Were excluded patients in intensive care units, palliative care and ventilated within 24 hours of admission. Three analyses were performed to adjust for selection bias: propensity score matching, multivariable survival, and inverse probability score weighting (IPSW) analyses.

**Results:**

Overall, 203 patients were included: 60 patients treated by HCQ/AZI and 143 control patients. During the 28-days follow-up, 32 (16.3%) patients presented the primary outcome and 23 (12.3%) patients died. Propensity-score matching identified 52 unique pairs of patients with similar characteristics. In the matched cohort (n = 104), HCQ/AZI was not associated with the primary composite outcome (log-rank p-value = 0.16). In the overall cohort (n = 203), survival and IPSW analyses also found no benefit from HCQ/AZI. In the HCQ/AZI group, 11 (18.3%) patients prolonged QT interval duration, requiring treatment cessation.

**Conclusions:**

HCQ/AZI combination therapy was not associated with lower in-hospital mortality and mechanical ventilation rate, with a 28-days follow-up. In the HCQ/AZI group, 18.3% of patients presented a prolonged QT interval requiring treatment cessation, however, control group was not monitored for this adverse event, making comparison impossible.

## Introduction

During the first few months after the coronavirus disease-2019 (COVID-19) outbreak, chloroquine and its derivative hydroxychloroquine (HCQ) have been suggested as treatment [1]. These molecules showed antiviral activity against the coronavirus in-vitro [2], although molecular mechanisms were not been fully explained, pH change on cell surface membrane has been suggested as a mechanism which may inhibit their fusion with COVID-19 [3]. A small non-randomized single-center study yielded moderate biological benefit in patients treated by HCQ combined with azithromycin (HCQ/AZI) compared to control group, with a 6-days follow-up [4]. Its mains limitations were numerous: small sample (36 patients), no clinical endpoint, and a fourth of HCQ/AZI patients lost to follow-up.

Even though numerous randomized and non-randomized controlled trials yielded negative results since then [5–7]; during the first semester of 2020, many healthcare centers started to use HCQ/AZI for COVID-19 hospitalized patients in accordance with a decree of the French government, despite the lack of clear data demonstrating the effectiveness of the HCQ/AZI combination [8].

Using data extracted from a single-center regional hospital, we hereafter describe the effects of such treatment on mortality and need for mechanical ventilation in patients hospitalized for COVID-19 pneumonia, during the first wave pandemic in France. We also assessed the incidence of QT prolongation, a known side effect of HCQ/AZI treatment combination [9].

## Methods

### Study design

This study is an observational retrospective cohort study based on registry which included all patients hospitalized for COVID-19 at Montfermeil community hospital, a secondary care center, located in Paris area with 670 beds and an ICU.

Patients were screened for eligibility if they satisfied the following criteria: adult patients with ward admission for COVID-19 pneumonia confirmed by real-time reverse transcription-PCR (RT-PCR) on a nasopharyngeal swab and/or typical imaging characteristics on chest computed tomography (CT) scan. Study period was March 15th, 2020 to April 2nd, 2020. They were then excluded if they required invasive mechanically ventilation (i.e. requiring orotracheal intubation) or were transferred in intensive care medicine department within the first 24 hours, were recused for intensive care due to futility, or died within the first 24 hours.

The study was approved by the Research Ethics Committees (Comité de Protection des Personnes “SUD-EST IV” number 20.04.21.83855) and by our local ethic committee in accordance with French legislation on non-interventional studies. Study protocol conforms to the ethical guidelines of the 1975 Declaration of Helsinki as reflected in a priori approval by the institution’s human research committee. Written informed consent was waived by the Ethics Committee given the observational nature of the study. This study is ancillary to the Adverse Events Related to Treatments Used Against Coronavirus Disease 2019 (COVIDTox) study (registered on clinicaltrials.gov with NCT04314817).

### Exposure

Patients were compared in two groups: those who received HCQ/AZI (HCQ/AZI group) and those who did not (control group). In the HCQ/AZI group, treatment was started within the first 24 hours as part of local protocol. They received 200 mg three times per day hydroxychloroquine for 10 days, and 500 mg once for azithromycin, on the first day. Azithromycin was then administered with 250 mg once per day for 4 days. The decision to administrate HCQ/AZI combination was taken for each patient individually by medical team at bedside. Pregnant women were systematically excluded from this treatment combination.

### Study outcomes

Main outcome was a composite of mortality and need for invasive mechanical ventilation (i.e. which required orotracheal intubation), during the first 28 days after hospital admission.

Tolerance was assessed by monitoring QT interval prolongation in the HCQ/AZI group. This safety protocol involved 12 lead electrocardiograms monitoring on day 1, 2, 3, 6, 9 and 12. Treatment was stopped in patients with corrected QT duration by Bazett’s or Framingham formulae more than 450 ms for men and 460 ms for women after first intake [10, 11].

### Follow up and data collection

Patients discharged from hospital were routinely called for follow up. Follow up was complete for all patient for in hospital mortality and 96.6% at 28 days.

All variables were collected as electronical chart research form in a prospective registry, during the study. Variables which were prospectively collected included: first symptoms date, hospitalization date, COVID-19 confirmation date, age, sex, body-mass index (BMI), history of diabetes, hypertension, asthma, chronic obstructive pulmonary disease (COPD), heart failure, active cancer, long-term corticosteroids, immunosuppressive therapy, non-steroidal anti-inflammatory drugs (NSAID), institutionalization, HCQ/AZI treatment during hospitalization, in-hospital mortality, need for intensive care unit transfer and need for mechanical ventilation. Variables which required secondary extraction from electronical charts included smoking status, betablockers, angiotensin-converting enzyme inhibitors (ACEI) or angiotensin receptor blockers (ARBs), antialdosterone, oxygen flow and saturation at admission, systolic and diastolic blood pressure, heart rate, Glasgow coma scale (GCS) and respiratory rate on admission, blood gas with P/F ratio, C-reactive protein (CRP), leucocytes count, platelets count, creatininemia, total bilirubin and Sequential Organ Failure Assessment (SOFA) score at admission.

### Statistical analyses

Data distribution was assessed using Shapiro Wilk test. Continuous variables were summarized as mean (standard deviation (SD)) and compared using a Student’s t-test. Continuous variables which were not normally distributed were compared using Wilcoxon-Mann-Whitney tests. Categorical variables were summarized as number (proportion) and compared using a chi-square test and Fischer exact test when appropriate. Baseline characteristics of patients in the HCQ/AZI group were compared to that of the control group. HCQ/AZI and control groups were compared in intention-to-treat. Because all patients with HCQ/AZI were treated within the first 24 hours of admission, first day of analysis was considered the day of admission.

In the primary analysis, 1:1 nearest-neighbor propensity-score matching was performed using the following variables: age, sex, smoking status, body-mass index, diabetes, hypertension, AIEC or ARB treatment, COPD, P/F ratio, SOFA score, CRP, leucocytes count, creatininemia and total bilirubin at admission. A second analysis using a multivariable Cox proportional hazard analysis was performed in the entire cohort of included patients, using covariables selected after univariate comparison between patients who presented the outcome and those who did not. A third analysis using inverse probability score weighting (IPSW) approach was also performed using the entire dataset [12, 13].

Briefly, each patient was weighted using the inverse of propensity score for patients on HCQ/AZI, and inverse of (1-propensity score) for those in the control group. The effect of HCQ/AZI on clinical outcomes was assessed in the weighted inflated population. This method aims to mitigate non-randomized allocation of treatment and was used previously in other retrospective studies including one in COVID-19 setting [14–16]. Kaplan-Meier curves were used to compare HCQ/AZI group and control group on time to the first outcome with a censorship at 28 days. Cox proportional hazards modeling was used to assess the association between HCQ/AZI and the primary outcome. After determining relevant independent variables from univariate comparison between HCQ/AZI group and control group, retained variables which were entered in the Cox survival model were age, O2 requirement at admission and creatininemia. List of available data is presented in S1 File; no data was imputed. All analyses were performed using SPSS v25.0 (IBM, Armonk, USA) and **Fig 2** was generated using R software (R project, worldwide community project).

## Results

**Fig 1:**
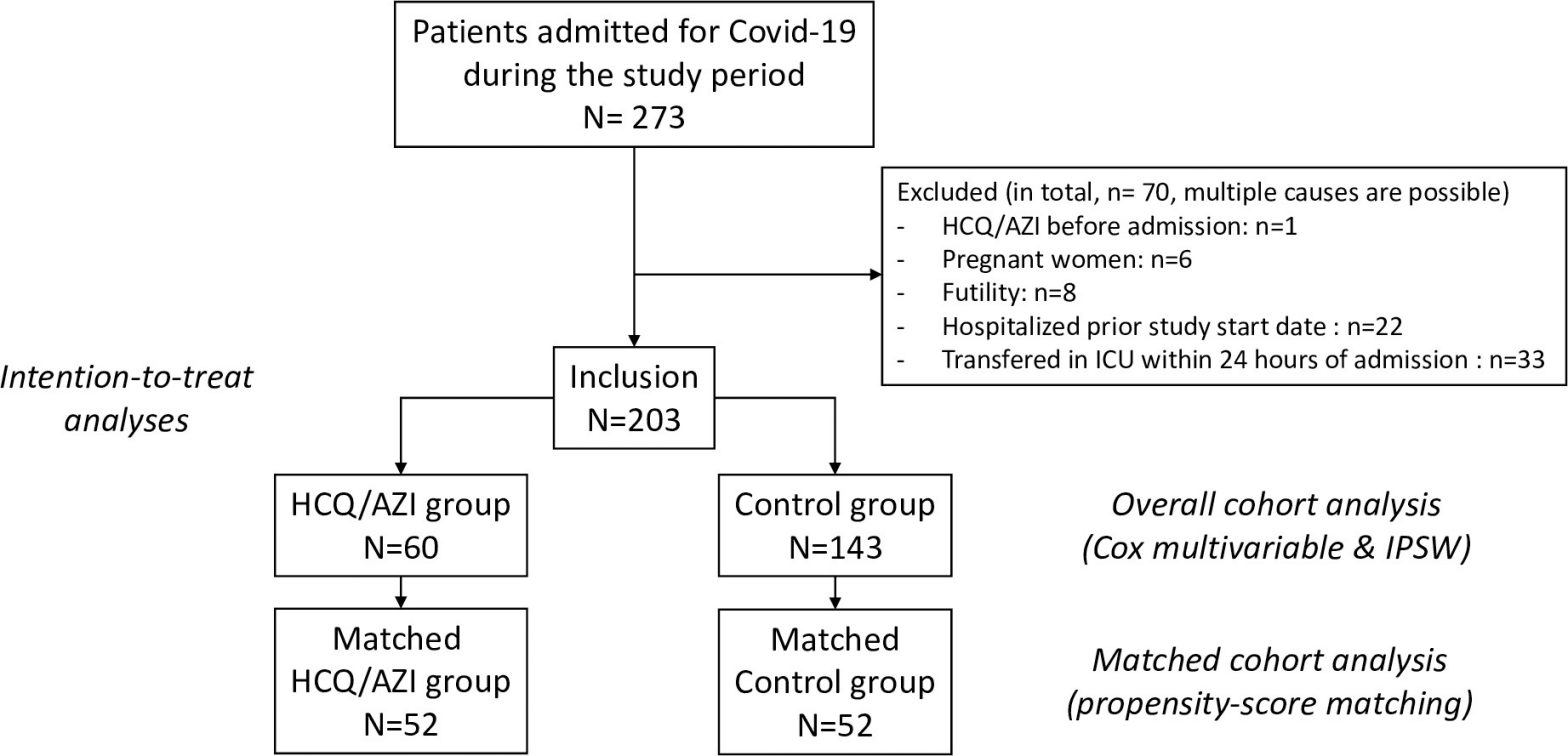
Study flow-chart. Abbreviations: HCQ/AZI: Hydroxychloroquine combined with azithromycin; IPSW: Inverse probability score weighted.

Flow-chart is presented in **Fig 1**. In total, 203 patients were included, among whom 60 patients treated by HCQ/AZI. Baseline characteristics are presented in **Table 1**. In the overall cohort (n = 203), patients who underwent treatment with HCQ/AZI were younger, there were proportionally more men, presented higher BMI, fewer heart failure, but were more severe at admission with a higher SOFA score and oxygen support requirement. Delay between first symptoms and hospital admission was similar in both groups, with a mean of 7.0 ±4.0 days. During the 28-days censored follow-up, 33 (16.3%) patients presented the primary outcome (mechanical ventilation or death). Overall, 25 (12.3%) patients died during follow-up.

**Table 1:** Demographic and clinical characteristics of the patients at baseline. Unless noted otherwise, parametric comparisons were performed.

		Overall (n = 203)	Control Group (n = 143)	HCQ/AZI Group (n = 60)	*P value*
**Demographic and clinical data**					
Age—years		59.6 ± 16.8	62.8 ± 17.3	52.1 ± 13.0	<0.0001
Male sex		120 (59.1)	77 (53.8)	43 (71.7)	0.02
BMI—kg/m2		29.0 ± 6.2	28.9 ± 6.3	30.8 ± 5.2	0.04 §
**Comorbidities, No (%)**					
Hypertension		90 (44.3)	72 (50.3)	18 (30.0)	0.01
Diabetes		67 (33.0)	53 (37.1)	14 (23.3)	0.06
Active smoking		7 (3.4)	6 (4.2)	1 (1.7)	0.53 ɸ
Past smoking		22 (10.8)	13 (9.1)	9 (15.0)	
Chronic heart failure		17 (8.4)	17 (11.9)	0 (0)	0.01
COPD		13 (6.4)	8 (5.6)	5 (8.3)	0.53 ɸ
Asthma		16 (7.9)	8 (5.6)	8 (13.3)	0.09 ɸ
Cancer		9 (4.4)	8 (5.6)	1 (1.7)	0.29 ɸ
**Treatment, No (%)**					
ACEIs or ARBs		50 (24.8)	39 (27.5)	11 (18.3)	0.17
Betablocker		41 (20.3)	34 (23.9)	7 (11.7)	0.05
Antialdosterone		5 (2.5)	4 (2.8)	1 (1.7)	1 ɸ
NSAIDs or corticoid		4 (2.0)	4 (2.8)	0 (0)	1 ɸ
**Admission data, mean ± SD in unit**					
Time from symptom onset to admission—days		6.5 ± 4.5	6.2 ± 4.0	7.2 ± 5.4	0.26 §
GCS		15 ± 0.1	15 ± 0.2	15 ± 0	0,19 §
Respiratory rate—/min		27.7 ± 8.4	26.7 ± 8.2	30.0 ± 8.3	0.01 §
Oxygen saturation—%		94.0 ± 4.0	94.1 ± 4.0	93.5 ± 3.9	0.17 §
Oxygen flow at admission—L/min		3.0 ± 3.2	2.9 ± 3.5	3.3 ± 2.4	0.01 §
P/F ratio		275.6 ± 106.2	283.1 ± 107.7	258.8 ± 101.6	0.03§
Systolic blood pressure—mmHg		132.9 ± 20.0	132.5 ± 21.6	134.1 ± 15.5	0.61
Heart rate—bpm		91.0 ± 16.8	90.3 ± 17.9	92.7 ± 13.7	0.35
SOFA score		2.3 ± 1.3	2.2 ± 1.3	2.6 ± 1.3	0.04 §
**Biology at admission mean ± SD in unit**					
C-reactive protein—mg/l		98.0 ± 74.8	98.9 ± 76.3	96.0 ± 71.7	0.86§
Leukocyte count—G/l		7.0 ± 3.1	6.9 ± 3.2	7.1 ± 2.9	0.59 §
Lymphocyte count—G/l		1.2 ± 0.6	1.2 ± 0.6	1.3 ± 0.5	0.37 §
Creatinemia—μmol/l		102.1 ± 79.5	109.0 ± 92.8	85.6 ± 23.1	0.36 §

Abbreviations: HCQ/AZI, Hydroxychloroquine/Azithromycin; BMI, body mass index; COPD, Chronic obstructive pulmonary disease; ACEIs, angiotensin-converting enzyme inhibitors; ARBs, angiotensin receptor blockers; NSAIDs Nonsteroidal Anti-Inflammatory Drugs; GCS, Glasgow Coma Scale; SOFA score, Sequential Organ Failure Assessment score.

§ refers to non-parametric comparisons.

ɸ refers to Fisher exact test.

### Matched cohort analysis

**Fig 2:**
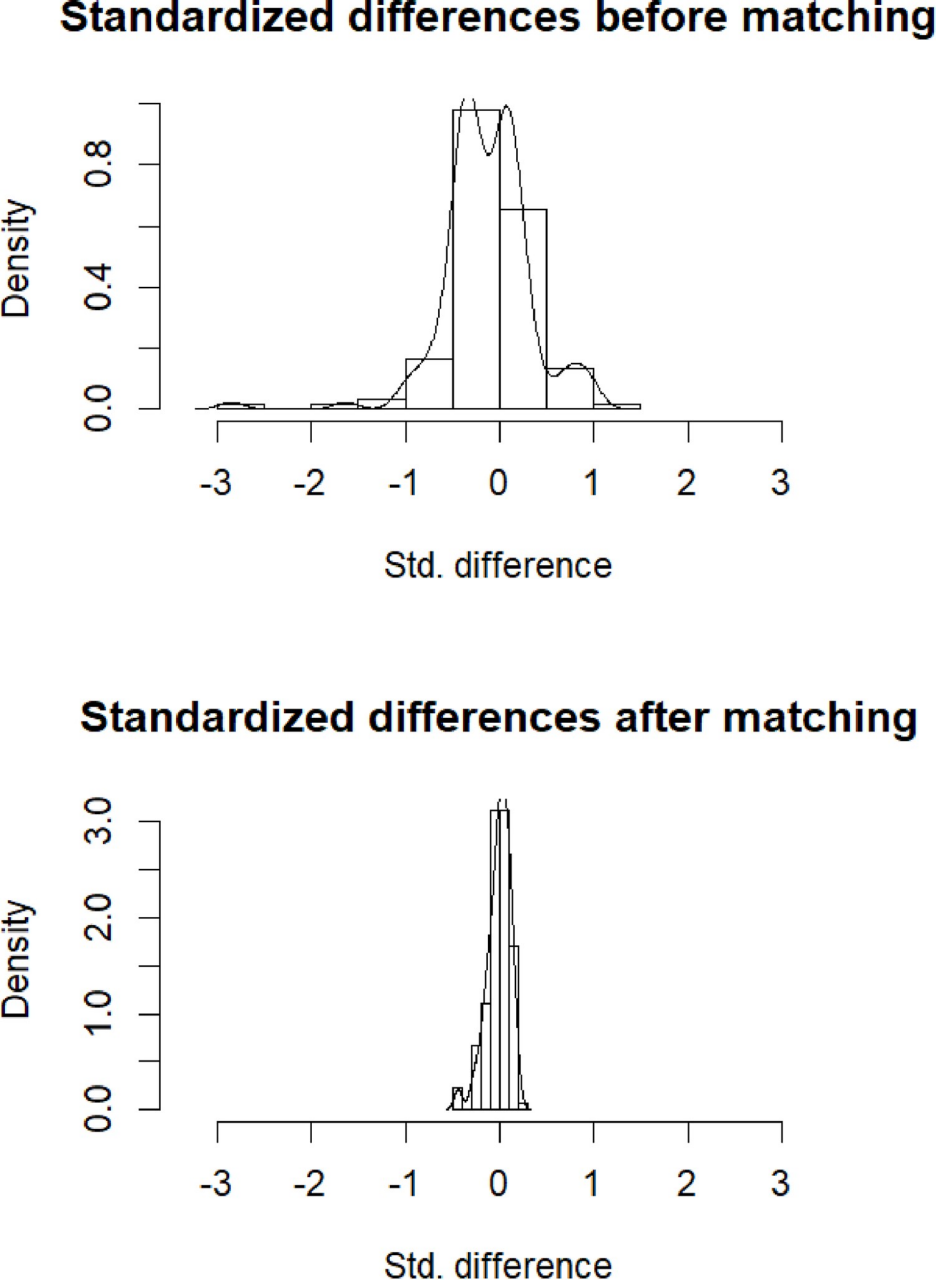
Standardized differences between HCQ/AZI and control group, before (A) and after (B) propensity-score matching.

Propensity-score matching found 52 pairs of patients (i.e. n = 104) in the matched cohort (baseline characteristics comparisons are presented in **Table 2)**. Matching was adequate: no above-mentioned differences in the overall cohort were significant in the matched cohort (see **Table 2** and **Fig 2**). In the matched cohort (n = 104), 13 patients presented the primary outcome (9 (17.3%) versus 4 (7.7%) in the control group, Chi-2 p = 0.24). Comparing survival curves, HCQ/AZI was not associated with the primary outcome (log-rank p-value = 0.16).

**Table 2:** Demographic and clinical characteristics of matched cohort patients. Unless noted otherwise, parametric comparisons were performed.

	Matched control (n = 52)	Matched HCQ/AZI group (n = 52)	*P value*
**Demographic and clinical data**			
Age—years	53.1 ± 14.8	53.4 ± 12.3	0.92
Male sex	36 (69.2)	35 (67.3)	0.83
BMI—kg/m2	30.1 ± 5.8	31.1 ± 5.1	0.36 §
**Comorbidities, No (%)**			
Hypertension	20 (38.5)	19 (36.5)	0.84
Diabetes	20 (38.5)	13 (25.0)	0.14
Active smoking	2 (3.8)	0 (0)	0.44 ɸ
Past smoking	5 (9.6)	7 (13.5)	
Chronic heart failure			
COPD	4 (7.7)	3 (5.8)	1 ɸ
Asthma	1 (1.9)	7 (13.5)	0.06 ɸ
Cancer	1 (1.9)	1 (1.9)	1 ɸ
**Treatment, No (%)**			
ACEIs or ARBs	13 (25.0)	11 (21.2)	0.64
Betablocker	9 (17.3)	8 (15.4)	0.79
Antialdosterone	3 (5.8)	1 (1.9)	0.62 ɸ
NSAIDs or corticoid			
**Admission data, mean ± SD in unit**			
Time from symptom onset to admission—days	7.2 ± 4.1	7.2 ± 5.7	0.70 §
GCS	15 ± 0.1	15 ± 0	0.32 §
Respiratory rate—/min	26.0 ± 6.8	30.4 ± 8.6	0.006 §
Oxygen saturation—%	93.9 ± 4.2	92.9 ± 5.2	0.21 §
Oxygen flow at admission—L/min	3.3 ± 3.6	3.1 ± 2.2	0.31 §
P/F ratio	257.0 ± 88.0	263.4 ± 106.1	0.67 §
Systolic blood pressure—mmHg	128.3 ± 21.5	136.1 ± 15.2	0.04
Heart rate—bpm	91.1 ± 14.7	92.3 ± 12.8	0.67
SOFA score	2.5 ± 1.2	2.5 ± 1.2	0.71§
**Biology at admission mean ± SD in unit**			
C-reactive protein—mg/l	111.1 ± 81.2	98.0 ± 78.7	0.36 §
Leukocyte count—G/l	6.9 ± 3.6	7.1 ± 3.0	0.58§
Lymphocyte count—G/l	1.2 ± 0.5	1.2 ± 0.5	0.53 §
Creatinemia—μmol/l	93.7 ± 65.2	87.2 ± 25.2	0.57 §

Abbreviations: HCQ/AZI, Hydroxychloroquine/Azithromycine; BMI, body mass index; COPD, Chronic obstructive pulmonary disease; ACEIs, angiotensin-converting enzyme inhibitors; ARBs, angiotensin receptor blockers; NSAIDs Nonsteroidal Anti-Inflammatory Drugs; GCS, Glasgow Coma Scale; SOFA score, Sequential Organ Failure Assessment score.

§ refers to non-parametric comparisons.

ɸ refers to Fisher’s exact test.

### Overall cohort analysis

**Fig 3:**
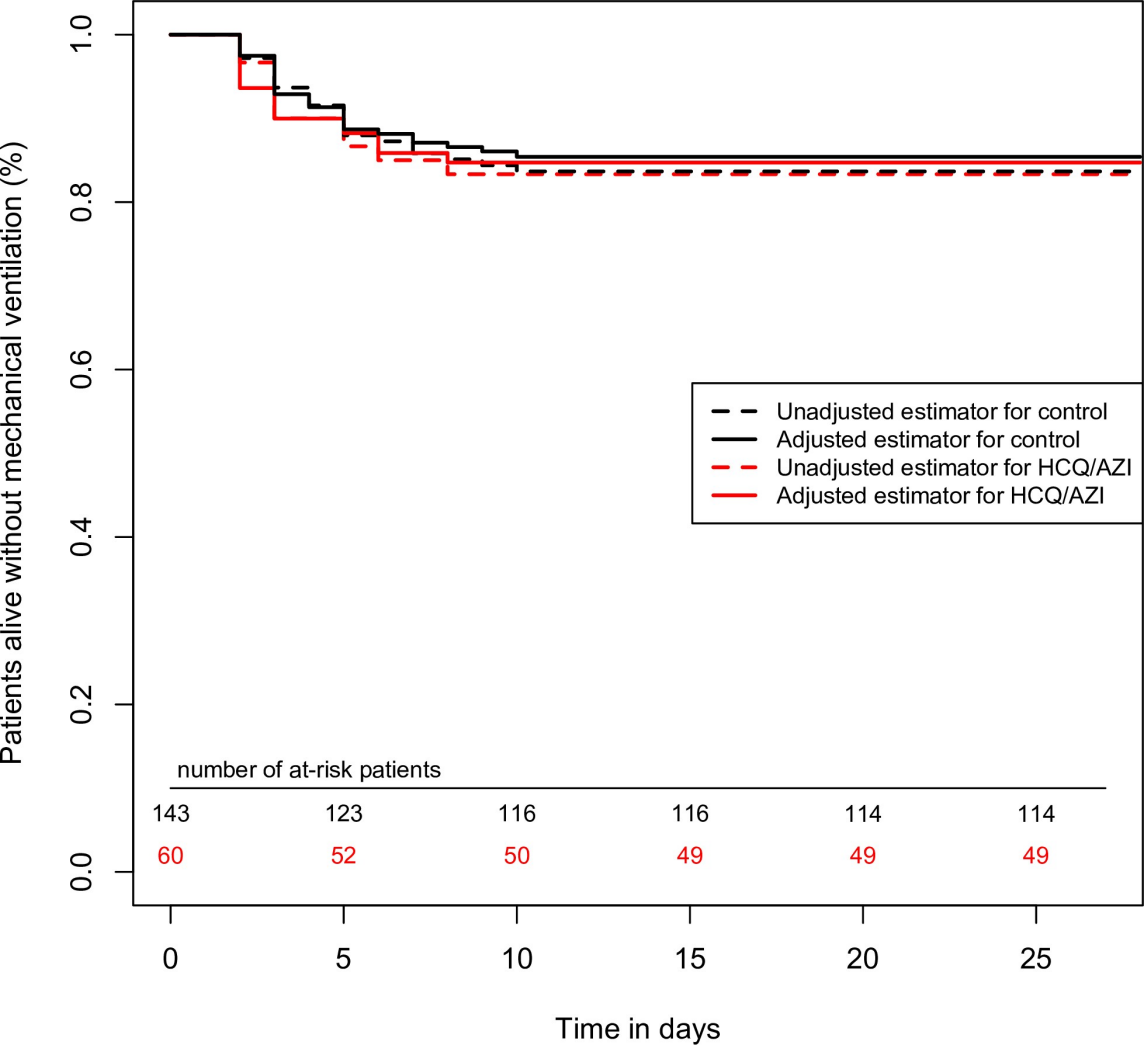
Survival curves, regarding the primary outcome, comparing HCQ/AZI and control group. In the propensity-score matched cohort (n = 203), HCQ/AZI was not associated with the primary outcome (p = 0.16). In the overall cohort (n = 104), there was no association in univariate survival comparison, nor in inverse probability score weighted analysis (p = 0.79 for both).

In the overall cohort (n = 203), in univariate analysis, HCQ/AZI was not associated with a reduction of the primary endpoint (unadjusted hazard ratio = 1.03 (95%CI = 0.49–2.17), p-value = 0.93). Cox multivariable survival analysis showed that variables associated with the primary composite endpoint were: age (per 1-year increase, adj.HR = 1.04 (95%CI = 1.01–1.06), p-value = 0.002), oxygen requirement at admission (per 1-L/min increase, adj.HR = 1.10 (95%CI = 1.05–1.22), p-value = 0.001) and creatininemia (per 10-micromol/L increase, adj.HR = 1.04 (1.02–1.06), p-value<0.0001). IPSW found similar results, with a lack of association between HCQ/AZI and the primary outcome with p-value = 0.79 (**Fig 3)**.

### Safety

In the group treated with HCQ/AZI, 11 (18.3%) of patients had an enlargement of QT interval duration, requiring treatment cessation. No ventricular tachyarrhythmia nor torsade de pointes occurred during the study period.

## Discussion

In this observational retrospective analysis comparing patients hospitalized for COVID-19 treated with HCQ/AZI combination therapy to patients who were not, we did not observe any difference in survival without mechanical ventilation, with a 28-days follow-up.

The cohort we described was consistent with previously reported cohorts of patients hospitalized for COVID-19. Indeed, there were proportionally more men [17], patients presented cardiovascular risk factors such as hypertension and diabetes [18]. Overall 28-days mortality was 12.3%, consistent with previous reports [19], and accounting for the fact we excluded patients who were admitted directly to intensive care medicine departments. Variables independently associated with the primary outcome were age, higher oxygen requirement at admission and creatininemia, all of which were also reported before [20].

Several studies yielded similar results. In chronological order, large observational cohorts showed a lack of benefit of HCQ. Geleris et al published a single-center observational study in 1376 in-patients with COVID-19 of whom 58.9% received HCQ, and found no association between HCQ and intubation or death (hazard ratio = 1.04, 95% (CI = 0.82 to 1.32) [21]. However, treatment duration was limited to 5 days shorter than recommended by other authors [22], and did not specifically study the HCQ/AZI combination with AZI-treated patients in both groups. Similarly, Mahevas et al. described in a French multicenter observational study of 181 COVID-19 patients who required oxygen, HCQ treatment did not affect survival without acute respiratory distress syndrom compared to control group (hazard ratio = 1.3 (95%CI = 0.7 to 2.6) [23]. Rosenberg et al. published a multicenter retrospective observational study, aggregating data from 25 New York metropolitan region hospitals and 1438 COVID-19 inpatients. They concluded to no benefit of HCQ/AZI on in hospital mortality with an alarming signal: HCQ/AZI was independently associated with cardiac arrest (hazard ratio 2.13, 95% CI, 1.12 to 4.05) [24].

More recently, two randomized controlled trials confirmed the lack of efficacy of HCQ with or without AZI. In the RECOVERY trial, a national multicenter study which included patients from 176 hospitals in the United Kingdom, 1561 patients treated with HCQ were compared with 3155 patients treated with usual care. Enrollment stopped due to lack of efficacy, and rate ratio of death was 1.09 (0.97–1.23), p = 0.15. There was even a signal towards an increased risk of mechanical ventilation in patients not under mechanical ventilation at baseline (risk ratio 1.14 (1.03–1.27)). In this study, QT prolongation was not assessed in all patients [25].

In another randomized controlled trial comparing HCQ/AZI in patients with mild-to-moderate COVID-19 pneumonia, 667 patients were randomized, among whom 504 had confirmed COVID-19 pneumonia. Similarly, there was no difference between patients treated with HCQ/AZI and those without (odds-ratio = 0.99 (0.57–1.73), p = 1.00). In this study, QT interval was prolonged in 14.7% of patients treated by HCQ/AZI, while only in 1.7% in the control group (the difference was statistically significant) [26].

Finally, in the international Solidarity trial, 11,330 patients were randomized to receive a treatment aimed against COVID-19, and 954 patients were treated by HCQ. Rate ratio of in-hospital mortality, assessed at day 28, was 1.19 (0.89–1.59), p = 0.23, which also was not in favor of using this treatment against COVID-19 [7].

Hence, in our study, we confirmed all the elements: we did not observe any benefit of HCQ/AZI, even with a longer follow-up of 28-days. More importantly, as supporting previous observations of cardiotoxicity with HCQ/AZI, we observed QT interval duration enlargement in 18.3% in the HCQ/AZI group. Trained cardiologists closely monitored QT interval enlargement, emphasizing the between-specialists-collaboration required in this pandemic. This high prevalence of QT interval enlargement may be explained by the restrictive criteria chosen leading to treatment cessation and close ECG monitoring. This restrictive criterion was chosen in a context of lack of clinical proof of benefit from treatment and known adverse events associated with HCQ/AZI.

Yet, HCQ, like chloroquine, blocks IKr and several cases of prolonged QT and torsades de pointes were associated with this drug, albeit after longer exposure [27, 28]. Likewise, AZI was associated with prolonged QT interval and risk of sudden death [29, 30]. A plausible synergistic effect of the disease with a proarrythmogenic effect of the inflammatory syndrome and cytokines may also participate to this rate of long QT [9, 31, 32].

We acknowledge several limitations. First, the retrospective study design inherently presents selection bias. To mitigate it, patients who were at extremely high risk, such as those who were admitted directly to ICU and those who deteriorated in the first 24 hours, were systematically excluded. Hence, only patients eligible for HCQ/AZI treatment were retained. Moreover, we performed a triple statistical analysis, involving propensity-score matching, multivariable Cox survival and IPSW. While we aimed to mitigate selection bias, this methodology may not replace a full-fledged randomization. Second, the single-center nature of the study requires external validation, although the baseline characteristics presented by the cohort and the incidence of mortality are similar to that of previously published cohorts. Third, although patients were prospectively included with prospective data collection, a few variables were collected retrospectively with very few missing values. To mitigate for this bias, main outcome was a composite of hard clinical endpoints. Fourth, we acknowledge the study may have been underpowered to detect a significant difference, due to the number of patients and the follow-up duration. However, as observed in the survival curves, there was no difference between the two groups, with curves crossing each other starting on day 5, and even a disadvantage for HCQ/AZI group in the matched cohort, making a superiority of HCQ/AZI in hospitalized patients unlikely. Finally, due to study design, patients in the control group were not systematically controlled with EKG, thus we cannot affirm that the QT interval enlargement observed was not exclusively due to COVID 19, as it may have been due to inflammation and dyskaliemia. However, these findings are comforted by adequately sized randomized controlled trials which were published between time of patients’ inclusion and time of publication.

## Conclusion

In this retrospective study including patients hospitalized for COVID-19 pneumonia, HCQ/AZI combination therapy was not associated with lower 28 days mortality and mechanical ventilation rate. In the HCQ/AZI group, 18.1% presented a prolonged QT interval that required treatment cessation.

## Supporting information

S1 File (XLSX)
